# Magnetic In_*x*_Ga_1 - *x*_N nanowires at room temperature using Cu dopant and annealing

**DOI:** 10.1186/1556-276X-10-3

**Published:** 2015-01-07

**Authors:** Youn Ho Park, Ryong Ha, Tea-Eon Park, Sung Wook Kim, Dongjea Seo, Heon-Jin Choi

**Affiliations:** Department of Materials Science and Engineering, Yonsei University, Seoul, 120-749 Republic of Korea; Spin Convergence Research Center, Korea Institute of Science and Technology (KIST), Seoul, 136-791 Republic of Korea

**Keywords:** InGaN, Nanowires, Diluted magnetic semiconductors (DMS), Copper dopant, Annealing

## Abstract

Single-crystal, Cu-doped In_*x*_Ga_1 - *x*_N nanowires were grown on GaN/Al_2_O_3_ substrates via a vapor-liquid-solid (VLS) mechanism using Ni/Au bi-catalysts. The typical diameter of the Cu:In_*x*_Ga_1 - *x*_N nanowires was 80 to 150 nm, with a typical length of hundreds of micrometers. The as-grown nanowires exhibited diamagnetism. After annealing, the nanowires exhibited ferromagnetism with saturation magnetic moments higher than 0.8 μ_B_ (1 μ_B_ × 10^-24^ Am^2^) per Cu atom at room temperature by the measurements using a superconducting quantum interference device (SQUID) magnetometer. X-ray absorption and X-ray magnetic circular dichroism spectra at Cu *L*_2,3_-edges indicated that the doped Cu had a local magnetic moment and that its electronic configuration was mainly 3*d*^9^. It possessed a small trivalent component, and thus, the n-type behavior of electrical property is measured at room temperature.

## Background

In addition to manipulating the charges of electrons, spintronic devices also control the spins of electrons. This innovative technique promises to be one of the next-generation concepts that will lead to the replacement of conventional devices. Recently, not only bulks but also one- and two-dimensional channels are considered as candidates for developing spintronic devices [1–3]. Several III-V compound nanowires exhibit characteristic advantages for this application. Both of the strong spin-orbit interactions induced from inversion asymmetry in wurtzite or zinc blende structure and one-dimensional structures have a potential of high efficiency for spin transport.

To develop spintronic devices, ferromagnetic materials must be used as an electrode. The main principle underlying the operation of spintronic devices is that the spin-polarized current is injected from a ferromagnetic electrode to the semiconductor channel and detected by placing a ferromagnetic detector on the other side of the channel. However, due to the lattice mismatch between the ferromagnet and the semiconductor, the spin injection efficiency is insufficient. Semiconductors doped with a transition metal, the so-called diluted magnetic semiconductors (DMSs), are promising candidates for resolving this problem. According to the principle of mean field theory [4], transition metals such as Sc, Ti, V, Cr, Mn, Fe, Co, and Ni that have partially filled *d* states can be doped for transforming spin-frustrated semiconductors into ferromagnets [5–7]. However, these transition metals with local magnetic moments have some limitations when acting as the doping elements because magnetic secondary clusters have been found to be ferromagnetic [8–10].

In this study, we investigated Cu-doped In_*x*_Ga_1 - *x*_N nanowires. In the previous study, we investigated Cu-doped GaN nanowires and found evidence of ferromagnetism [11]. Cu is a non-magnetic element; however, if it is doped into a semiconductor in such a way that it is in a divalent state, the partially filled *d* states of the Cu^+2^ ions render it a candidate for ferromagnetism [11]. Meanwhile, the In_*x*_Ga_1 - *x*_N compound semiconductor has the accessibility of band gap modulation, which carries a high potential for wide-ranging applications. If magnetism is observed in Cu-doped In_*x*_Ga_1 - *x*_N nanowires, it is the first observation of evolution of magnetism in In-Ga-N semiconductor system by doping of a non-magnetic element. It suggests band gap tunable, magnetic In-Ga-N semiconductors that could be versatile DMSs toward spintronics.

## Methods

Single In_*x*_Ga_1 - *x*_N nanowires were synthesized using Ni/Au bi-metal catalysts deposited on sapphire (c-Al_2_O_3_) substrates in a horizontal hot-wall chemical vapor transport system. The thicknesses of the Ni and Au were 0.5 and 2 nm, respectively. Between the bi-metal catalysts and the sapphire substrates, a 30-nm single-crystalline GaN film was inserted to reduce lattice mismatch. Trimethylgallium (TMGa), trimethylindium (TMIn), and ammonia flowed as precursor gases, thereby supplying the sources of indium and gallium. CuCl powder (purity 99.99%) was inserted into the center of a quartz tube at intervals of 2 in. The substrates were loaded into a reactor and heated to the growth temperature of 700°C under flows of 100 sccm H_2_ and 100 sccm N_2_. The TMGa, TMIn, and NH_3_ were then allowed to flow for 10 min. Some nanowires were annealed at 800°C by using a rapid thermal annealing process. The composition of In_*x*_Ga_1 - *x*_N nanowires could be modulated with the range 0 ≤ *x* ≤ 0.51 that the band gap energies correspond from 2.17 to 3.1 eV by adjusting the amount of TMGa and TMIn and the growth temperatures. Among these, the In_*x*_Ga_1 - *x*_N nanowires with *x* = 0.09 that corresponds to the band gap energy of 3.1 eV were chosen for this experiment. The concentration of Cu in these nanowires was analyzed as 1.8%.

The nanowires were characterized using transmission electron microscopy (TEM) and energy-dispersive spectroscopy (EDS). The magnetic properties of the nanowires on the substrate were measured in a superconducting quantum interference device (SQUID) magnetometer, with corrections being made to take into account the diamagnetic contribution from the substrate. Anomalous X-ray scattering (AXS) and X-ray magnetic circular dichroism (XMCD) measurements were carried out at the 2A beamline of the Pohang Light Source (PLS). For the XMCD measurement, the degree of circular polarization of the incident light was set to be 95%, and a 0.5-T magnetic field that is produced by an electromagnet was applied along the surface normal to the sample to align the spin moment.

For electric measurement, the nanowires were sonicated in ethanol and dispersed to SiO_2_/p-doped Si substrate. We deposited a Ti/Au electrode of 70 nm on one In_*x*_Ga_1 - *x*_N nanowire channel using conventional photo-lithography and a sputtering system. A 300-nm SiO_2_ oxide layer and a highly p-doped Si layer were used for the bottom gate insulator and gate electrode, respectively.

## Results and discussion

**Figure 1:**
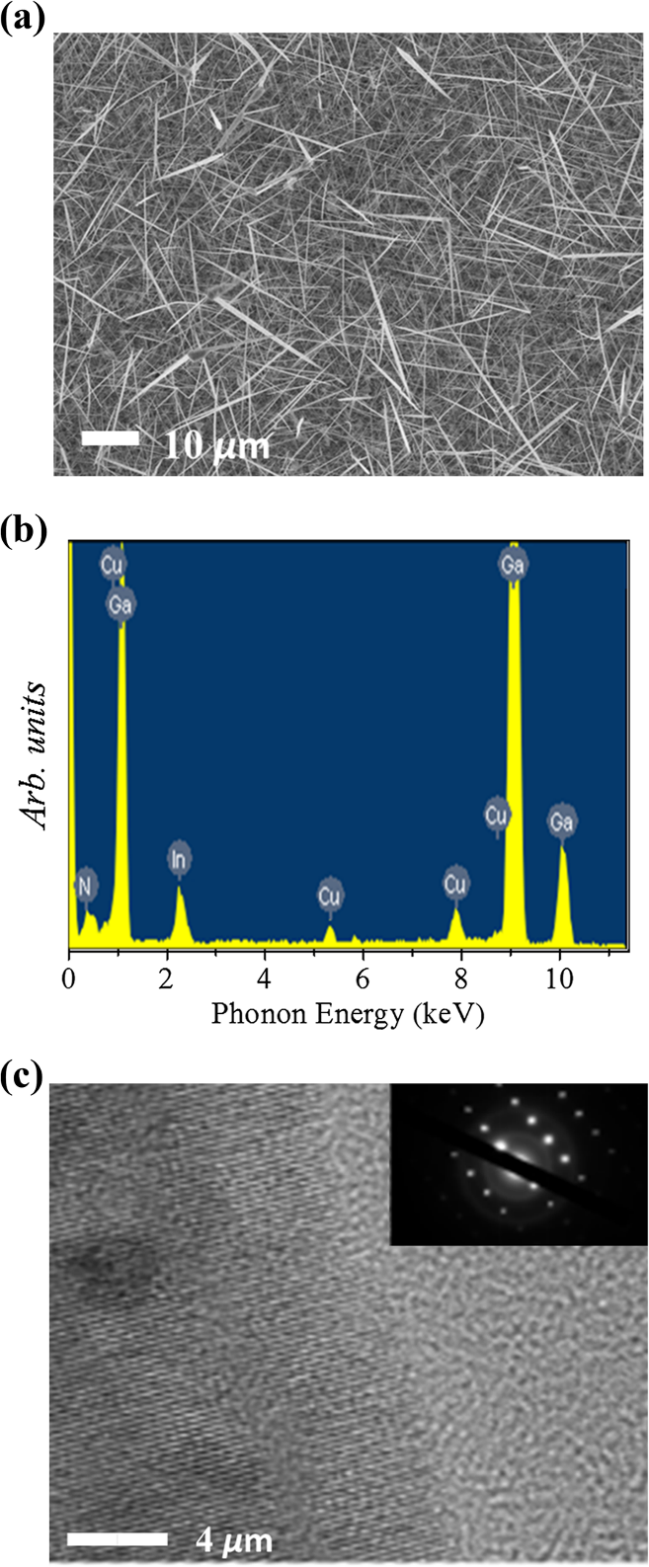
**SEM, EDS, and HRTEM images and SAED pattern of nanowires. (a)** Typical SEM image of Cu-doped In_*x*_Ga_1 - *x*_N nanowires. **(b)** EDS image of the nanowire. **(c)** HRTEM image and the SAED pattern (inset) of an 80-nm-diameter nanowire.

Figure 1a shows a scanning electron microscopy (SEM) image of the Cu-doped In_*x*_Ga_1 - *x*_N nanowires grown on the substrate with Au/Ni as bi-catalyst [12]. The nanowires have diameters in the range of 80 to 150 nm and lengths in the range of hundreds of micrometers. The nanowires were sonicated in ethanol and dropped to molybdenum grids for the EDS and TEM analyses. EDS was used to determine the composition of Cu in the Cu-doped In_*x*_Ga_1 - *x*_N nanowires. Figure 1b shows the respective Cu concentrations at the center of the Cu-doped In_*x*_Ga_1 - *x*_N nanowire. The average Cu concentration was about 1.8%. Figure 1c shows a high-resolution TEM image, which illustrates that the nanowires are single crystals without defects and secondary phases. In the inset of Figure 1c, the selected area electron diffraction (SAED) pattern shows the nanowires grown along the [001] direction. We also characterized the annealed nanowires and found any structural and compositional changes in the nanowires.

**Figure 2:**
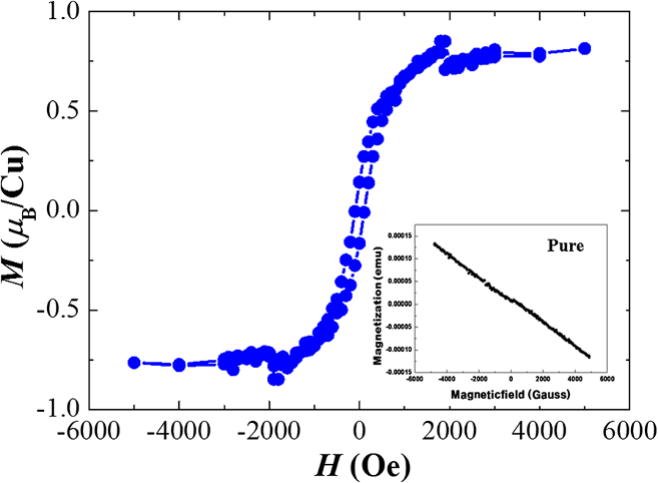
**Magnetic properties after rapid thermal annealing under a flow of N**_**2**_ **.** The inset shows the magnetic properties of pure In_*x*_Ga_1 - *x*_N nanowires, indicating diamagnetism.

Figure 2 shows the field dependence of magnetization for Cu-doped In_*x*_Ga_1 - *x*_N nanowires, which was measured with a SQUID magnetometer at room temperature and after rapid thermal annealing (800°C). The inset of Figure 2 shows the magnetic properties of the as-grown In_*x*_Ga_1 - *x*_N nanowires, which indicate diamagnetism at room temperature. This is due to the fact that Cu may be interstitially doped into In_*x*_Ga_1 - *x*_N nanowires, since this type of doping is more energetically favorable than substitution doping [13–15]. In order to resolve this issue, the Cu dopants in the In_*x*_Ga_1 - *x*_N nanowires were activated through rapid thermal annealing under a flow of N_2_ gas. It is known that annealing dissociates Cu by occupying the interstitial sites and substituted Ga or In vacancies in the InGaN lattice. This leads to an increase in the hole concentration, which is essential for the evolution of ferromagnetism in the doped semiconductors [16–18]. As expected, we observed the clear hysteresis loops after rapid thermal annealing (800°C) even at room temperature, which indicates that nanowires possess ferromagnetism with a Curie point exceeding room temperature [18]. The magnetization increases steeply at a low magnetic field and saturates at about >0.15 T. The magnetic moment for Cu-doped In_*x*_Ga_1 - *x*_N nanowires in Figure 2 is shown to be 0.8 μ_B_ (1 μ_B_ × 10^-24^ Am^2^) per Cu atom at room temperature.

**Figure 3:**
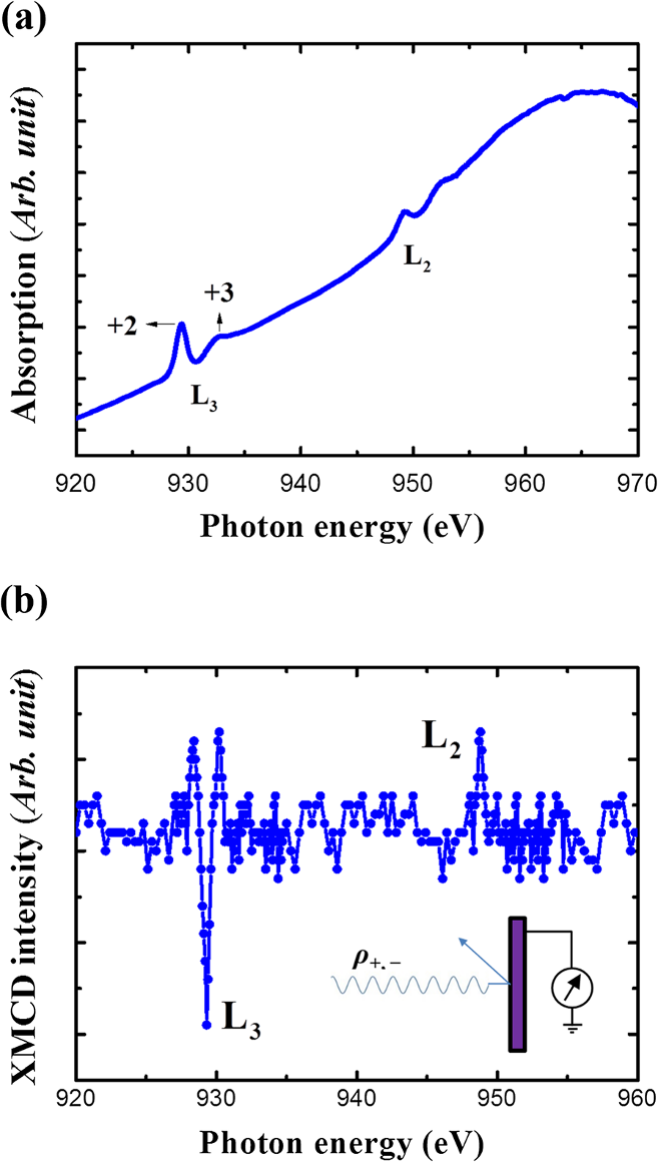
**AXS spectra at the Cu** ***L***_**2,3**_ **-edge and XMCD data. (a)** AXS spectra at the Cu *L*_2,3_-edge for Cu-doped In_*x*_Ga_1 - *x*_N measured at 300 K. The inset shows the AXS spectra at the Cu *L*_2,3_-edge for reference CuO powder. **(b)** XMCD data showing the difference between the Cu *L*_2,3_-edge AXS spectra for the different spin directions (*ρ*_+_ and *ρ*_-_) of nanowires measured at 300 K.

To investigate whether Cu dopants are incorporated in the crystalline lattice of In_*x*_Ga_1 - *x*_N nanowires or not, we measured AXS for the In_*x*_Ga_1 - *x*_N nanowires around the Cu K absorption edge. Figure 3a shows the *L*-edge absorption spectra for Cu-doped In_*x*_Ga_1 - *x*_N nanowires. The absorption spectrum peaks are separated into two regions of *L*_3_ and *L*_2_, due to the spin-orbit split of the core levels. This is consistent with the well-known 930- to 931-eV peak of CuO corresponding to the 3*d*^9^ ground state (2*p* to 3*d* dipole transition) [11]. The Cu absorption spectrum peak and the largest peak for Cu-doped In_*x*_Ga_1 - *x*_N nanowires are located at the same energy of 930 eV for the 3*d*^9^ ground state of CuO. There is also another identical but smaller peak at an energy level that is 3.5 eV higher. There is no such corresponding peak in divalent (2+) or monovalent (1+) Cu compounds [19, 20]. It is common in the AXS spectra that higher valence states of 3*d* transition metals appear at energies that are higher by 2 to 4 eV because of the decreased Coulomb energy between the core hole and valence electrons of the final state [21–23]. Accordingly, we expected that it is related to the trivalent (3+) state. The identical line structure is reproduced at the *L*_2_ region [19, 20]. It is thus confirmed that the electronic configuration of doped Cu is mainly 3*d*^9^. The formal ionic valence of Cu at cation sites is trivalent; however, it seems that the locally divalent state is preferred for covalent bonding with nitrogen. This suggests that the ionocovalent bonding nature of the Cu 3*d* orbital with the surrounding semiconductor medium provides the Cu atom with a mixed electron configuration.

If such a case occurs, then the spins of the 3*d* orbital are not paired and the doped Cu should have a local spin magnetic moment. To check whether the local spin moments align ferromagnetically or not, the XMCD spectra of Cu *L*_2,3_ absorption edges were also measured. As shown in Figure 3b, the dichroism signal was measured successfully. The theoretical multiplet spectrum for the 3*d*^9^ configuration in a wurtzite crystal field is reproduced. Consequently, it is proven that the doped Cu provides a local magnetic moment, and this is the cause for the ferromagnetism of the Cu-doped In_*x*_Ga_1 - *x*_N nanowires at room temperature. In this system, no XMCD dichroism signal at the Ni *L*_2,3_-edge in the samples was observed, such as in the metallic or secondary phase of the Ni catalyst employed in the growth of the nanowire. This further suggests that the nanowires are ferromagnetic at room temperature and that the induced host moments are clearly associated with the ferromagnetic phase in the Cu-doped In_*x*_Ga_1 - *x*_N. This also provides compelling evidence against any role in the origin of ferromagnetism of the secondary phase and impurity.

We observed the Cu 3*d* state and its four nearest neighboring N 2*p* states to understand the mechanism that stabilizes the ferromagnetic state in the Cu-doped In_*x*_Ga_1 - *x*_N nanowire system [24]. In the majority spin channel, the Cu 3*d* overlaps with the four nearest neighboring N 2*p* states at the CuN_4_ tetrahedron with reduced magnetization; additionally, in this channel, the 2*p* state of the four connecting N atoms contributes significantly to the unoccupied states. These characteristics indicate strong hybridization between Cu and its four neighboring N atoms. This strong hybridization induces finite magnetization of the Cu atom as well as the neighboring N atoms. The induced magnetic moments generated by the delocalized Cu ion indicate that a long-range *p*-*d* exchange interaction can occur in the Cu-doped In_*x*_Ga_1 - *x*_N nanowire system. Indeed, recent theoretical studies have shown that this system is a promising DMS possessing ferromagnetism, which can be explained in terms of the *p*-*d* hybridization mechanism, with a Curie temperature around 350 K.

**Figure 4:**
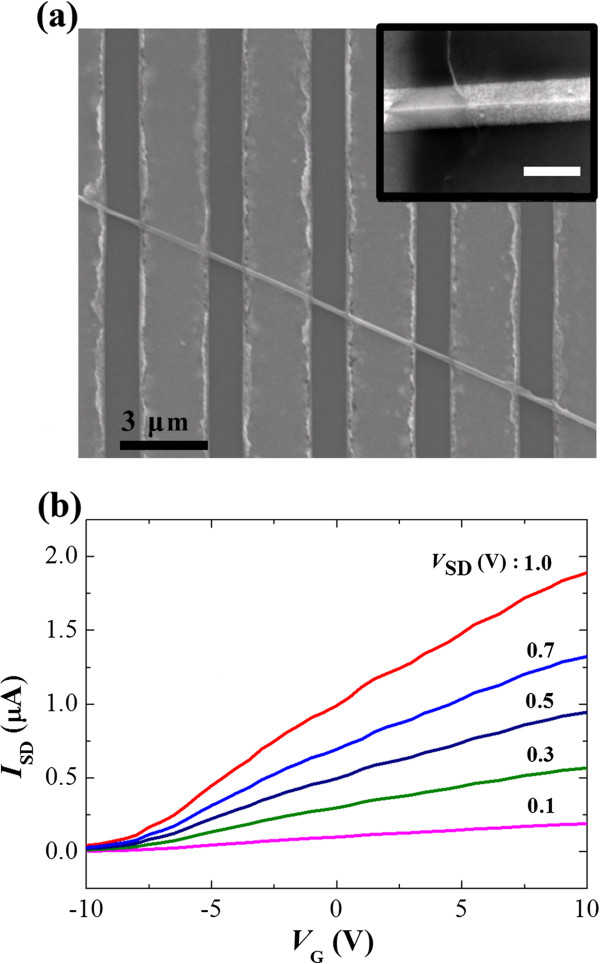
**Scanning electron micrograph and results of electrical measurement. (a)** Scanning electron micrograph of the electric device. The inset shows the close-up of the nanowire channel and the scale bar is 100 nm. **(b)** Results of electrical measurement with various *V*_SD_ at 300 K.

The annealing could make the nanowires increase the hole concentration due to dissociating Cu by occupying the interstitial sites and substituted Ga or In vacancies in the InGaN lattice. For investigating the electric properties, we fabricated a field-effect transistor using bottom gate voltage on one Cu-doped In_*x*_Ga_1 - *x*_N nanowire channel after the annealing process, as shown in Figure 4a. In the inset of Figure 4a, the close-up of the triangle nanowire channel and the diameter is about 120 nm, and the channel length is about 1.5 μm. Figure 4 shows the gate voltage dependence of the source-drain current (*I*_SD_) at various source-drain voltages (*V*_SD_), which indicates n-type behavior. This result is similar to those found in previous papers that reported n-type electrical behavior [11, 18], but these are inconsistent with the evolution of hole-mediated magnetism. The In_*x*_Ga_1 - *x*_N system including nanowires in this study has n-type electrical behavior due to the structural defects. In the system, Cu could act as p-type dopant if it fully substitutes Ga or In sites and ionized. In this study, however, the Cu dopants may not yet fully occupy the Ga or In sites in the In-Ga-N lattice by the annealing process, and thus, the n-type behavior was still observed although the magnetism was evolved by Cu.

## Conclusions

Single-crystal and homogeneous Cu-doped In_*x*_Ga_1 - *x*_N nanowires were fabricated and were shown to exhibit ferromagnetism at room temperature after rapid thermal annealing (800°C) under a flow of N_2_ gas. XMCD spectra of Cu *L*_2,3_ absorption edges were measured, and the dichroism signal was calculated successfully. It is proved that the doped Cu provides the local magnetic moment and is the origin of the ferromagnetism of the Cu-doped In_*x*_Ga_1 - *x*_N nanowires at room temperature. It is the first observation of the evolution of magnetism in In-Ga-N semiconductors by doping of a non-magnetic element. By considering the band gap tunable, direct band gap nature of In-Ga-N systems, such an evolution of magnetism suggests versatile DMSs toward spintronics.

## Abbreviations

AXS: anomalous X-ray scattering
DMS: diluted magnetic semiconductor
EDS: energy-dispersive spectroscopy
PLS: Pohang Light Source
SEM: scanning electron microscopy
SQUID: superconducting quantum interference device
TEM: transmission electron microscopy
XMCD: X-ray magnetic circular dichroism.
